# Above–Belowground Herbivore Interactions in Mixed Plant Communities Are Influenced by Altered Precipitation Patterns

**DOI:** 10.3389/fpls.2016.00345

**Published:** 2016-03-23

**Authors:** James M. W. Ryalls, Ben D. Moore, Markus Riegler, Scott N. Johnson

**Affiliations:** Hawkesbury Institute for the Environment, Western Sydney UniversityRichmond, NSW, Australia

**Keywords:** aboveground–belowground interactions, alfalfa, amino acids, aphids, climate change, grass–legume mixture, herbivores

## Abstract

Root- and shoot-feeding herbivores have the capacity to influence one another by modifying the chemistry of the shared host plant. This can alter rates of nutrient mineralization and uptake by neighboring plants and influence plant–plant competition, particularly in mixtures combining grasses and legumes. Root herbivory-induced exudation of nitrogen (N) from legume roots, for example, may increase N acquisition by co-occurring grasses, with knock-on effects on grassland community composition. Little is known about how climate change may affect these interactions, but an important and timely question is how will grass–legume mixtures respond in a future with an increasing reliance on legume N mineralization in terrestrial ecosystems. Using a model grass–legume mixture, this study investigated how simultaneous attack on lucerne (*Medicago sativa*) by belowground weevils (*Sitona discoideus*) and aboveground aphids (*Acyrthosiphon pisum*) affected a neighboring grass (*Phalaris aquatica*) when subjected to drought, ambient, and elevated precipitation. Feeding on rhizobial nodules by weevil larvae enhanced soil water retention under ambient and elevated precipitation, but only when aphids were absent. While drought decreased nodulation and root N content in lucerne, grass root and shoot chemistry were unaffected by changes in precipitation. However, plant communities containing weevils but not aphids showed increased grass height and N concentrations, most likely associated with the transfer of N from weevil-attacked lucerne plants containing more nodules and higher root N concentrations compared with insect-free plants. Drought decreased aphid abundance by 54% but increased total and some specific amino acid concentrations (glycine, lysine, methionine, tyrosine, cysteine, histidine, arginine, aspartate, and glutamate), suggesting that aphid declines were being driven by other facets of drought (e.g., reduced phloem hydraulics). The presence of weevil larvae belowground decreased aphid numbers by 30%, likely associated with a significant reduction in proline in weevil-treated lucerne plants. This study demonstrates how predicted changes to precipitation patterns and indirect interactions between herbivores can alter the outcome of competition between N-fixing legumes and non-N-fixing grasses, with important implications for plant community structure and productivity.

## Introduction

### Species interactions and climate change

Ecological communities form a network of directly and indirectly interacting species (Wootton, 1994; Polis, 1998). These networks are often categorized into tractable ecosystem components (e.g., food chains, aboveground–belowground interactions, plant–insect interactions, competitive interactions) used to elucidate the causal mechanisms that underpin species interactions, and community dynamics (Holt, 1997; Rudolf, 2012). Notably, plants are exploited by multiple root and shoot herbivores, which can interact indirectly through plant-mediated mechanisms. They have the capacity to influence one another by modifying the chemistry of the shared host plant (Blossey and Hunt-Joshi, 2003; Johnson et al., 2012). Changes in host plant nutrients (e.g., amino acid concentrations) often underpin interactions between aboveground and belowground herbivores, with knock-on effects on plant community structure and productivity (Johnson et al., 2013). In particular, altered rates of root exudation in response to herbivory can influence rates of nutrient mineralization and acquisition by neighboring plant species (Bardgett et al., 1999; Ayres et al., 2007). However, all trophic interactions, both direct and indirect, are specific to the species combinations involved (Kos et al., 2015) and may be altered by climate change (McKenzie et al., 2013). Such changes may propagate through communities and sway competitive advantages between species (Johnson et al., 2011; Barton and Ives, 2014; Jing et al., 2015). The incidence of extreme precipitation events, including droughts and floods, is expected to increase in many regions throughout the world, with a generally drier future predicted for south-eastern Australia (Chiew et al., 2011; Dai, 2011). Drought is one of the most important environmental stressors for plants, often altering root to shoot biomass ratios (Gargallo-Garriga et al., 2014) and sometimes altering plant N and amino acid concentrations (Girousse et al., 1996; Garten et al., 2009; Johnson et al., 2011). Excess water can also create anaerobic conditions around plant roots, affecting growth, and nutrient status, although in areas with sufficient drainage, increased water availability is likely to positively affect plant productivity. Nonetheless, effects of drought are of longer duration and are considered to have far more disruptive effects on plant–insect interactions (Pritchard et al., 2007).

### Grass–legume ecosystems

Ecosystem processes, including primary productivity and nutrient cycling, tend to increase in biologically diverse plant communities, especially in mixtures containing leguminous plant species (Hooper et al., 2005; Hatch et al., 2007). Most legumes have the ability to fix atmospheric N_2_ via their symbiotic relationship with rhizobial bacteria (Long, 1989). Rhizobial bacteria are accommodated by nodules on legume roots, the development of which is strongly affected by soil conditions including pH, nutrient availability, temperature, and moisture (Ramos et al., 2003; Ferguson et al., 2013). Drought, in particular, can negatively impact nitrogenase activity in legume nodules (Sprent, 1972), although the mechanisms remain unclear (Streeter, 2003; Nasr Esfahani et al., 2014). Benefits of mixed plant communities (e.g., enhanced resource acquisition) arise from species complementarity (i.e., resource niche differentiation) or from selection effects (i.e., mixed communities are more likely to harbor a dominant productive species; Loreau and Hector, 2001; Turnbull et al., 2013). Legumes, in particular, fix more atmospheric N_2_ when mixed with grasses than when planted in monocultures, allowing companion grass species to utilize plant-available N for production (Chmelíková et al., 2015). Hence, diverse communities can enhance primary production and increase soil fertility (Hatch et al., 2007). Compared with legume monocultures, grass–legume mixtures also show more efficient water utilization and improved ground cover, which reduces runoff and erosion (Ta and Faris, 1987; Humphries, 2012). Moreover, mixed plant communities in general are often more productive (Tilman et al., 2001) and experience reduced herbivore damage by diluting the availability of specific host plants (Root, 1973; Haddad et al., 2009; Fabian et al., 2012). Water stress may have important consequences for grass–legume ecosystems. In particular, grasses, with their shallow root systems, tend to be more sensitive to drought than legumes (Hayes et al., 2012) but less sensitive to waterlogging (Neal et al., 2009). Hence, changes in rainfall patterns may disrupt the ability of plant species to coexist in a mixture (Tow, 1993; Tow et al., 1997).

### Belowground herbivory

Root-feeding herbivores of legumes, such as *Sitona* weevils, have the potential to disrupt the interactions between grasses and legumes by altering the flow of N between plant species (Ta et al., 1986; Murray and Hatch, 1994). *Sitona* adults feed on the foliage of legumes while the larvae feed on and in the N-fixing root nodules and on roots as they develop (Aeschlimann, 1979; Arbab and McNeill, 2014). Larval feeding can enhance legume root exudation and soil N availability and result in the increased availability of N for neighboring grasses (Murray and Clements, 1998). Previous studies, for example, have demonstrated that root pruning and herbivory by *Sitona* spp. enhanced the direct transfer of N from white clover (*Trifolium repens* L.) to a neighboring grass species (*Lolium perenne* L.; Hatch and Murray, 1994; Murray and Hatch, 1994). *Sitona* larvae are consistently more damaging than foliar-feeding adults and peak larval densities are often driven by prevailing moisture conditions (Cantôt, 1979; Goldson et al., 1986; Goldson S. et al., 1988). While root nodule herbivory can be highly damaging to the legume, reducing nodulation and impairing N-fixation, legume root recovery can also be over-compensatory, producing higher numbers of nodules, and increasing N-fixation in response to herbivory (Quinn and Hall, 1992; Ryalls et al., 2013b).

### Aboveground herbivory

Root damage by weevil larvae is likely to indirectly affect aboveground phloem-feeding herbivores by altering phloem turgor pressure and/or through changes in phloem amino acid concentrations. Aphids, in particular, tend to perform better on plants with higher N and amino acid concentrations (Ponder et al., 2000; Karley et al., 2002; Nowak and Komor, 2010; Ryalls et al., 2015). Moreover, aphid performance on plants could be affected not only by the overall amino acid concentration, but also by the proportional composition of different amino acids (Mittler, 1967; Srivastava and Auclair, 1975; Pritchard et al., 2007). Masters et al. (1993) argued that root herbivory promotes aphid performance by impairing soil water and nutrient uptake, which reduces the relative water content of foliage and increases soluble N (e.g., amino acids) and carbohydrate concentrations in the phloem. In contrast, Ryalls et al. (2013b) suggested that negative effects of *Sitona discoideus* Gyllenhal on pea aphids (*Acyrthosiphon pisum* Harris) could arise through lower quality phloem sap from nodule damage specifically, or reduced phloem turgor, and increased sap viscosity (via impaired root function), which would make the phloem more difficult to access.

The majority of sap-sucking invertebrates, including aphids, respond negatively to host plant water stress, which may relate similarly to a decrease in turgor pressure and an increase in phloem sap viscosity (Raven, 1983; Huberty and Denno, 2004). Moreover, increases in plant N (Johnson et al., 2011) and amino acids (Hale et al., 2003) under drought do not necessarily benefit aphids due to these accompanying changes in phloem physiology (Aslam et al., 2012). Effects are likely to be contingent on a range of factors including plant functional group or whether species are growing alone or in competition with others (van der Putten et al., 2004; Johnson et al., 2011).

### Study system, objectives, and hypotheses

Using a model grass–legume mixture of Harding grass (*Phalaris aquatica* L.) and lucerne (otherwise known as alfalfa, *Medicago sativa* L.), this study addressed the effects of water stress (simulated drought and elevated precipitation) and root nodule herbivory by *S. discoideus* on both the plant–plant interactions between lucerne and Harding grass and on foliar-feeding pea aphids (*A. pisum*), one of the most damaging pests of lucerne [see Ryalls et al. (2013a) for review]. The long-term co-existence of lucerne and grass can be perilous, with one often failing to persist under competition with the other (Bishop and Gramshaw, 1977; Dear et al., 1999). Harding grass, one of the most persistent sown temperate perennial grasses in south-eastern Australia, is one species that complements lucerne and achieves a dynamic balance in a mixture with lucerne (Sherrell, 1984; McKenzie et al., 1990; Culvenor et al., 2007).

This study aimed to determine whether (i) water stress and insect herbivory on lucerne influenced plant growth and N dynamics in both lucerne and Harding grass and (ii) whether water stress and insect herbivory affected total and individual foliar amino acid concentrations in lucerne and, subsequently, the population growth of *A. pisum*. We hypothesized that: (i) drought and elevated precipitation would decrease and increase plant growth, respectively, although both would negatively impact plant N concentrations in general. Lucerne nodule herbivory by *S. discoideus* was predicted to cause N leakage into the soil, which would positively affect the productivity of the co-occurring grass species; (ii) the negative impacts of drought and weevil herbivory on nodulation and N acquisition would decrease foliar amino acid concentrations and/or phloem turgor pressure in lucerne, which would reduce aphid populations. Moreover, specific decreases in individual amino acid concentrations would cause aphid populations to decline.

## Materials and methods

### Rain-exclusion shelters

Rain-exclusion shelters (249 × 188 cm) located at the Hawkesbury campus of Western Sydney University (latitude −33.609396, longitude 150.737800), as described by Johnson et al. (2015), were used to exclude 100% of ambient rainfall from four mesocosms beneath each of 18 shelters. Mesocosm pots (41 × 41 × 31 cm) were arranged in a 2 × 2 formation and dug into the ground so that the rim of the pot was flush with the soil surface. Each of the 72 mesocosms was filled with the excavated soil, which was air-dried and sieved to < 4 mm. Removable mesh cages (34 × 34 × 36 cm) were fitted to each mesocosm to prevent escape of experimental insects or entry to non-experimental (free-living) insects. The cages were designed to maximize air movement and allow good light transmittance (Johnson et al., 2015).

### Insect cultures

*Acyrthosiphon pisum* cultures, originating from a single parthenogenetic adult female collected from a local lucerne field in Richmond, NSW in August 2014, were maintained on propagated lucerne plants at 26/18°C day/night on a 15L:9D cycle until required. Eggs produced from 30 sexually mature *S. discoideus* adults collected from the same field and reared under the same conditions were collected every 24 h and stored on damp filter paper at 4°C until required. Hatching success of 2-month old eggs was confirmed (>97% hatched within 1 week) by placing 200 eggs on 10 Petri dishes at 25°C.

### Experimental procedure

**Figure 1:**
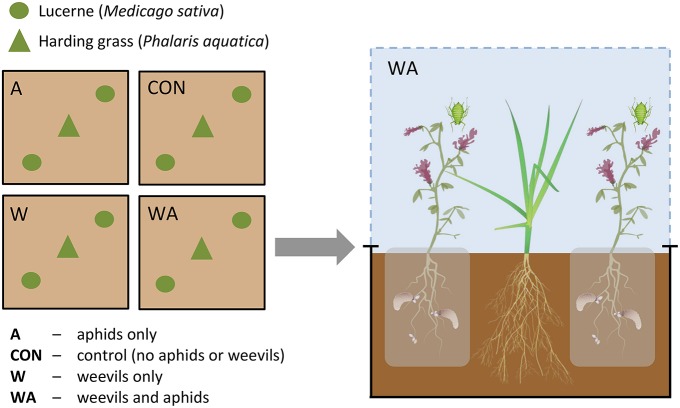
**Schematic diagram of experimental plots beneath one rain-exclusion shelter**.

Lucerne (cv. Sequel) seeds were inoculated with *Rhizobium* bacteria 1 h prior to sowing by submerging in a solution containing 250 g Nodule N lucerne seed inoculant (New Edge Microbials, Albury, NSW) and 800 mL distilled water. Lucerne seeds (*N* = 144) were sown and grown in seed cells (38 × 57 mm), with one lucerne plant per cell, under field conditions. Six grass seeds were sown in each of 72 seed cells and grown under the same conditions. After 4 weeks (1 October 2014), grass from one cell was transplanted into the center of each mesocosm and two lucerne plants were transplanted either side of the grass (see Figure 1). The root systems of the individual lucerne plants were contained within porous organza bags. This was done to confine weevil larvae to the soil around each lucerne plant, prevent their movement between plants and simplify inoculation with weevil eggs, but at the same time to not restrict transfer of water and nutrients within each mesocosm. Seed numbers within each mesocosm were based on a 1:1 lucerne:grass seed weight ratio (Boschma et al., 2010; Sandral, 2013).

Shelters were assigned at random to one of three rainfall treatments giving six shelters per rainfall regime. The ambient treatment was set at the 65.4 mm average precipitation in Richmond between September–November from 1881 to 2014 (Bureau of Meteorology, Australia). The drought treatment was 50%, and elevated precipitation treatment was 150% of the ambient rainfall amounts. This translated to an application of 586 mL (ambient precipitation), 293 mL (drought), and 878 mL (elevated precipitation) of rainfall water collected at the site three times per week. Soil moisture measurements were taken weekly using a 12 cm Hydrosense II probe (Campbell Scientific, Queensland, Australia).

Two weeks after transplantation, both lucerne plants in two of the mesocosms under each shelter were inoculated with 50 *S. discoideus* eggs, which were placed on top of the soil beside the stem of each plant. Egg numbers (i.e., 50 eggs per plant) were based on average densities of 4000 eggs and 80 lucerne plants per m^2^ (Aeschlimann, 1983; Goldson and French, 1983). After a further 2 weeks, three teneral *A. pisum* adults were applied to each of the two lucerne plants in one mesocosm containing *S. discoideus* and one mesocosm without *S. discoideus*. The factorial insect treatment design under each shelter therefore comprised one mesocosm with *S. discoideus* alone (W), one mesocosm with *A. pisum* alone (A), one mesocosm with both insects (WA), and one mesocosm with neither (CON; Figure 1).

*Acyrthosiphon pisum* individuals were counted and removed 4 weeks after being applied, whereupon the experiment was harvested. The number of plants that were colonized by aphids (i.e., aphid colonization success) were recorded. Grass tiller numbers and plant heights of both lucerne (from ground level to the base of the highest leaf) and Harding grass were measured six (weevil inoculation period), eight (aphid inoculation period), and 12 (harvest period) weeks after sowing. Roots were separated from the soil and shoots and the number of lucerne nodules were counted. Roots and shoots were frozen at −20°C, freeze-dried, and weighed prior to chemical analyses.

### C, N, and amino acid analyses

Freeze-dried root and leaf material was ball-milled to a fine powder and carbon (C) and nitrogen (N) concentrations of 4–6 mg of milled samples were determined using an elemental analyser (LECO TruSpec Micro, LECO Corp., St. Joseph, MI, USA). The N content of lucerne roots was calculated by multiplying root dry mass by the concentration of N in roots. Soluble amino acids were extracted and analyzed from milled lucerne foliage samples (15–20 mg) following the protocol set out by Ryalls et al. (2015). Foliar amino acids were used as a reliable and quantifiable proxy for the composition of phloem amino acids, as proposed by Riens et al. (1991) and Winter et al. (1992) for spinach and barley, respectively. Amino acid standards within the AA-S-18 (Fluka, Sigma-Aldrich) reference amino acid mixture were supplemented with asparagine and glutamine (G3126 and A0884, Sigma, Sigma-Aldrich). Nine essential amino acids (i.e., those that cannot be synthesized by insects *de novo*), including arginine, histidine, isoleucine, leucine, lysine, methionine, phenylalanine, threonine, and valine (Morris, 1991) and 10 non-essential amino acids (alanine, asparagine, aspartate, cysteine, glutamate, glutamine, glycine, proline, serine, and tyrosine) were detected using this method.

### Statistical analyses

Precipitation and herbivore treatment effects on plant growth (height, mass, grass tiller numbers, and lucerne nodulation), chemistry (carbon and nitrogen), and aphids (population numbers and colonization success) were analyzed using mixed models in the *nlme* (Pinheiro et al., 2014) and *lme4* (Bates et al., 2014) statistical packages within the R statistical interface v3.1.1. The fixed effects included herbivore treatment (CON, A, W, and WA) and precipitation (drought, ambient, and elevated precipitation) as well as the interactions between these terms. The random terms included shelter (1–18) as a block term, with plot number (i.e., positioning under each shelter) as a nested factor to account for shelter and plot effects that could confound any treatment effects. Plant heights measured at the three time-points (i.e., weeks 6, 8, and 12) were combined into an individual model incorporating time as a fixed effect to account for repeated measures. *Post-hoc* Tukey's tests using the *glht* function in the R package *multcomp* (Hothorn et al., 2008) and the R package *LMERConvenienceFunctions* (Tremblay and Ransijn, 2015) were used for pairwise comparisons of means for treatment and interaction effects. The effects of precipitation and herbivore treatment on all individual amino acid concentrations were determined using principal components analysis (PCA) and groupings of co-varying individual amino acids were determined using a correlation matrix and hierarchical clustering within R. Permutational analysis of variance (PERMANOVA) was used to determine the effects of precipitation and herbivore treatment on concentrations of correlated amino acid groups.

## Results

### Soil water

Soil water content was significantly affected by both precipitation and herbivore treatment, and their interaction (Table 1). Specifically, under ambient and elevated precipitation, soil water increased in plots containing weevils alone (Figure 2), although soil water did not increase significantly when both weevils and aphids were present.

**Table 1:** **Soil, plant, and aphid responses to precipitation and herbivore treatments from linear mixed models**.

**Response variable**	**Figures**	**Herbivore treatment**			**Precipitation**			**Herbivore treatment** × **Precipitation**		
		***F***	***P***	***df***	***F***	***P***	***df***	***F***	***P***	***df***
**SOIL CHARACTERISTICS**										
Soil water (%)^#^	2	16.10	**0.001**	3.45	223.00	<**0.001**	2.15	2.44	**0.039**	6.45
**PLANT CHARACTERISTICS**										
***Phalaris aquatica***										
Height (week 6)	3	1.91	0.141	3.45	0.35	0.708	2.15	0.58	0.741	6.45
Height (week 8)	3	2.41	0.080	3.45	28.09	<**0.001**	2.15	1.85	0.111	6.45
Height (week 12)	3	6.05	**0.002**	3.45	48.61	<**0.001**	2.15	1.38	0.245	6.45
Number of tillers	S1	3.12	**0.035**	3,45	84.22	<**0.001**	2,15	0.53	0.786	6.45
Shoot mass^#^	4	0.91	0.445	3.45	91.58	<**0.001**	2.15	1.67	0.149	6.45
Root mass^#^	4	2.24	0.097	3.45	21.15	<**0.001**	2.15	0.22	0.968	6.45
Shoot %N^*^	5	2.92	**0.044**	3.45	2.57	0.110	2.15	0.76	0.607	6.45
Root %N^*^	5	3.10	**0.036**	3,45	2.07	0.161	2,15	2.21	0.059	6.45
Shoot %C	S2	0.37	0.775	3.45	3.82	**0.046**	2.15	0.77	0.600	6.45
Root %C	S2	1.84	0.154	3.45	1.65	0.226	2.15	0.75	0.616	6.45
***Medicago sativa***										
Height (week 6)	3	0.47	0.702	3.45	0.08	0.926	2.15	0.52	0.793	6.45
Height (week 8)	3	0.69	0.565	3.45	3.90	**0.043**	2.15	0.31	0.926	6.45
Height (week 12)	3	1.67	0.187	3.45	70.57	<**0.001**	2.15	0.85	0.538	6.45
Shoot mass^*^	4	0.70	0.558	3.45	19.99	<**0.001**	2.15	0.47	0.827	6.45
Root mass^*^	4	1.51	0.226	3.45	9.44	**0.002**	2.15	0.50	0.801	6.45
Nodulation	6	4.38	**0.009**	3.45	3.84	**0.045**	2.15	0.26	0.952	6.45
Root %N	6	3.48	**0.024**	3.43	1.40	0.277	2.15	0.67	0.672	6.43
Root N content^*^	6	2.29	**0.092**	3.43	18.25	<**0.001**	2.15	1.02	0.423	6.43
Root %C	7	0.43	0.731	3.43	5.39	0.017	2.15	2.63	**0.029**	6.43
**APHID RESPONSES**										
Population^#^	9	4.56	**0.040**	1.33	5.38	**0.017**	2.15	2.14	0.134	2.33
Colonization	S5	3.35	0.076	1.33	3.73	0.048	2.15	3.32	**0.049**	2.33

P-values highlighted in bold indicate significance (P < 0.05). Where appropriate, response variables were transformed (

* *Log*,

# *Square root) before analysis*.

**Figure 2:**
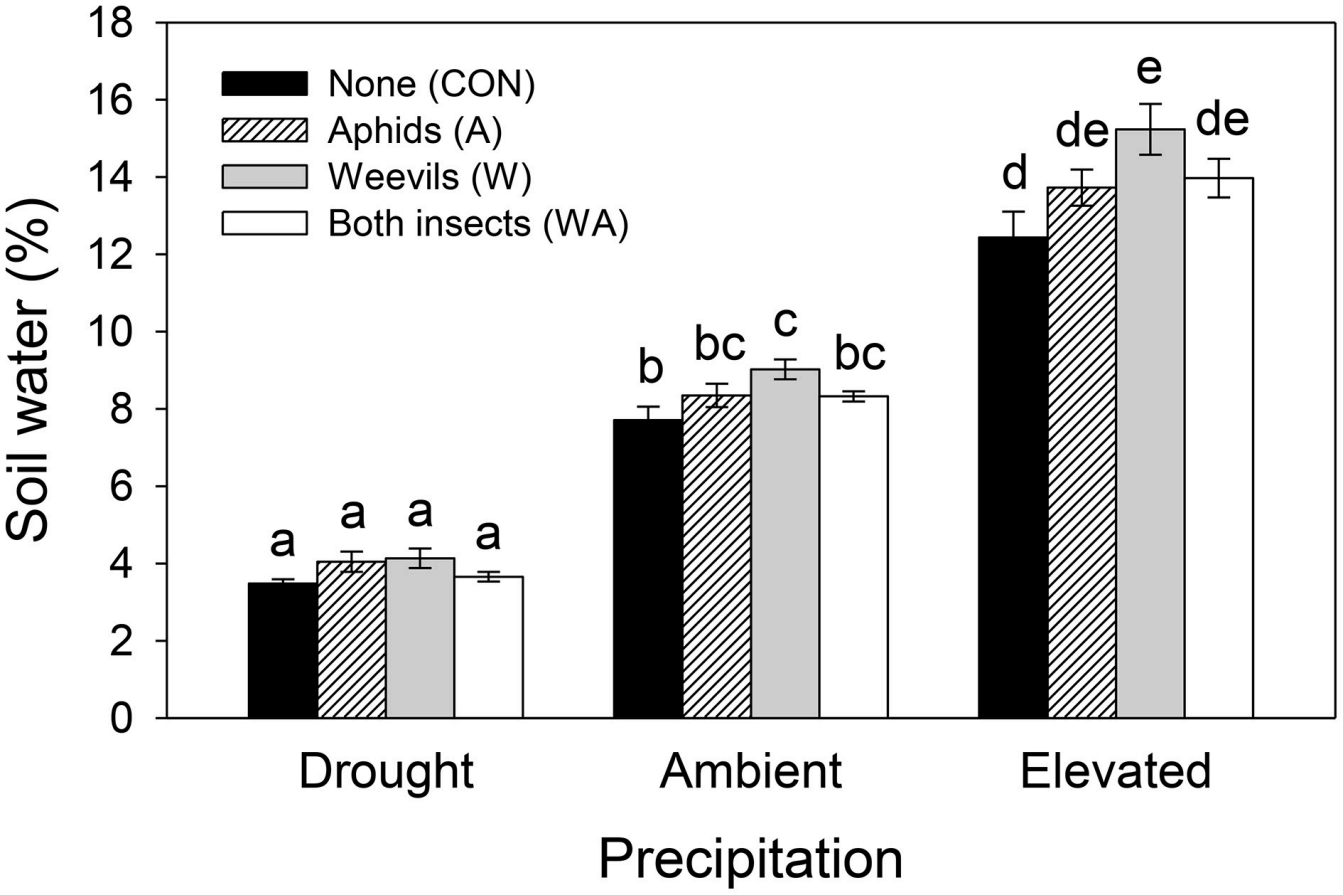
**The interactive effects of precipitation and herbivore treatment on soil water content**. Mean values (±SE) are shown. Bars with the same letters were not significantly different (*P* < 0.05).

### Plant growth

**Figure 3:**
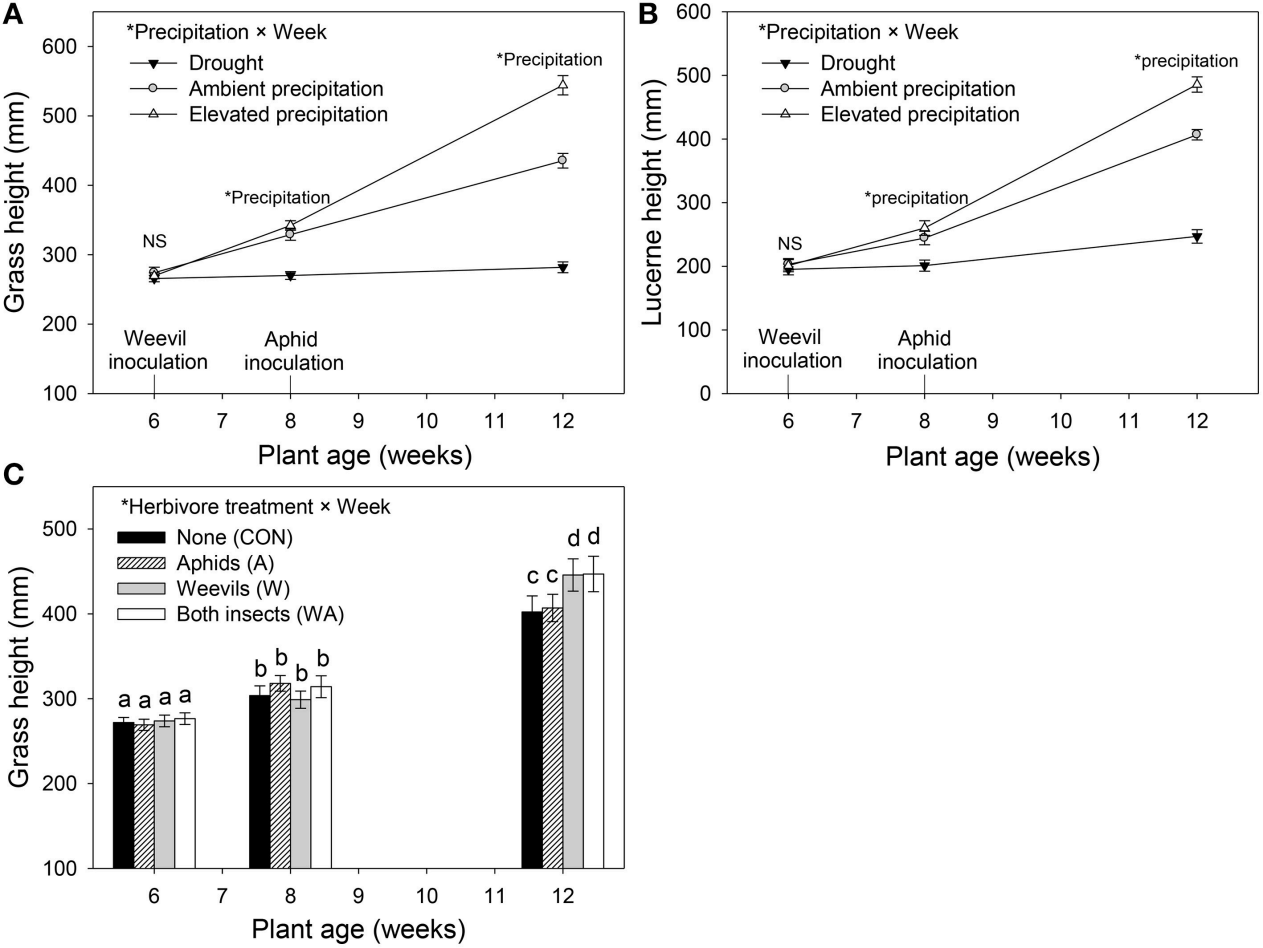
**The impacts of precipitation and herbivore treatment on the height of Harding grass (A,C) and lucerne (B) at week 6 (plant transplant period), week 8 (weevil inoculation period), and week 12 (harvest period)**. Herbivore treatment had no significant effect on lucerne height. Mean values (±SE) are shown. Statistically significant effects are indicated by ^*^(*P* < 0.05) and bars with the same letters were not significantly different (*P* < 0.05).

Precipitation affected both grass and lucerne height at week 8 and 12 (Table 1). Specifically, grasses and lucerne plants subjected to drought at week 8 were shorter than those grown under ambient and elevated precipitation, although height did not vary significantly between plants grown under ambient and those grown under elevated precipitation. By harvest (week 12), the height of both grasses and lucerne plants had increased with increasing precipitation (Figures 3A,B). Additionally, grasses were significantly taller in plots where lucerne plants had been inoculated with weevils (i.e., treatments W and WA; Figure 3C). For lucerne, herbivore treatment had no significant effect on height at any point (Table 1). Combining plant heights into individual models (accounting for repeated measures) revealed similar results (Table S1).

**Figure 4:**
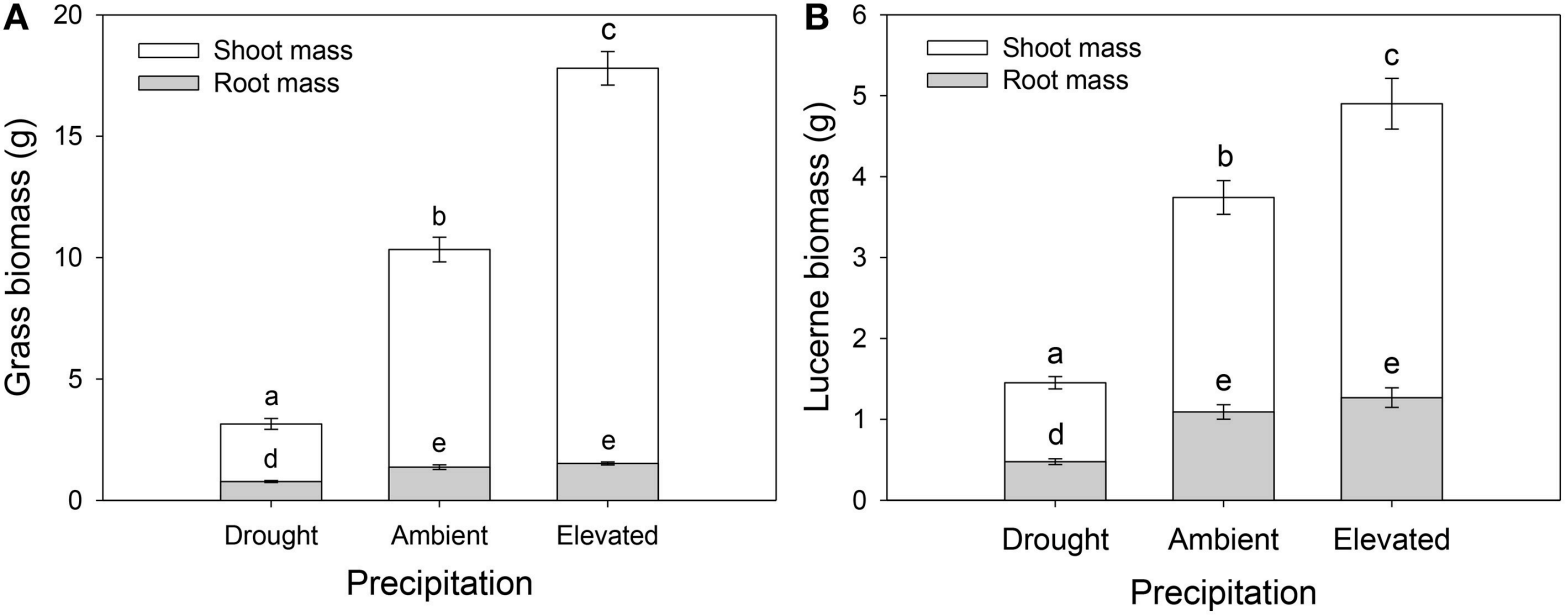
**The impacts of precipitation on the root and shoot biomass of Harding grass (A) and lucerne (B)**. Herbivore treatment had no significant effect on biomass. Mean values (±SE) are shown. Bars with the same letters were not significantly different (*P* < 0.05).

Grass tiller numbers increased with increasing precipitation (Figure S1A), with grasses grown under drought having, on average, 51 and 65% fewer tillers than those grown under ambient and elevated precipitation, respectively (Figure S1A). Moreover, grasses within plots inoculated with weevils alone contained 23% more tillers than those in control plots (Figure S1B). Grass and lucerne shoot mass increased with increasing precipitation (i.e., the biomass of grasses increased and decreased under elevated precipitation and drought, respectively, compared with those subjected to ambient precipitation), although root biomass did not vary significantly between those grown under ambient and elevated precipitation (Figures 4A,B). Root to shoot ratios of both plants decreased as water availability increased. Herbivore treatment had no effect on lucerne or grass biomass in general, individually or interactively (Table 1).

### Carbon, nitrogen, and nodulation

**Figure 5:**
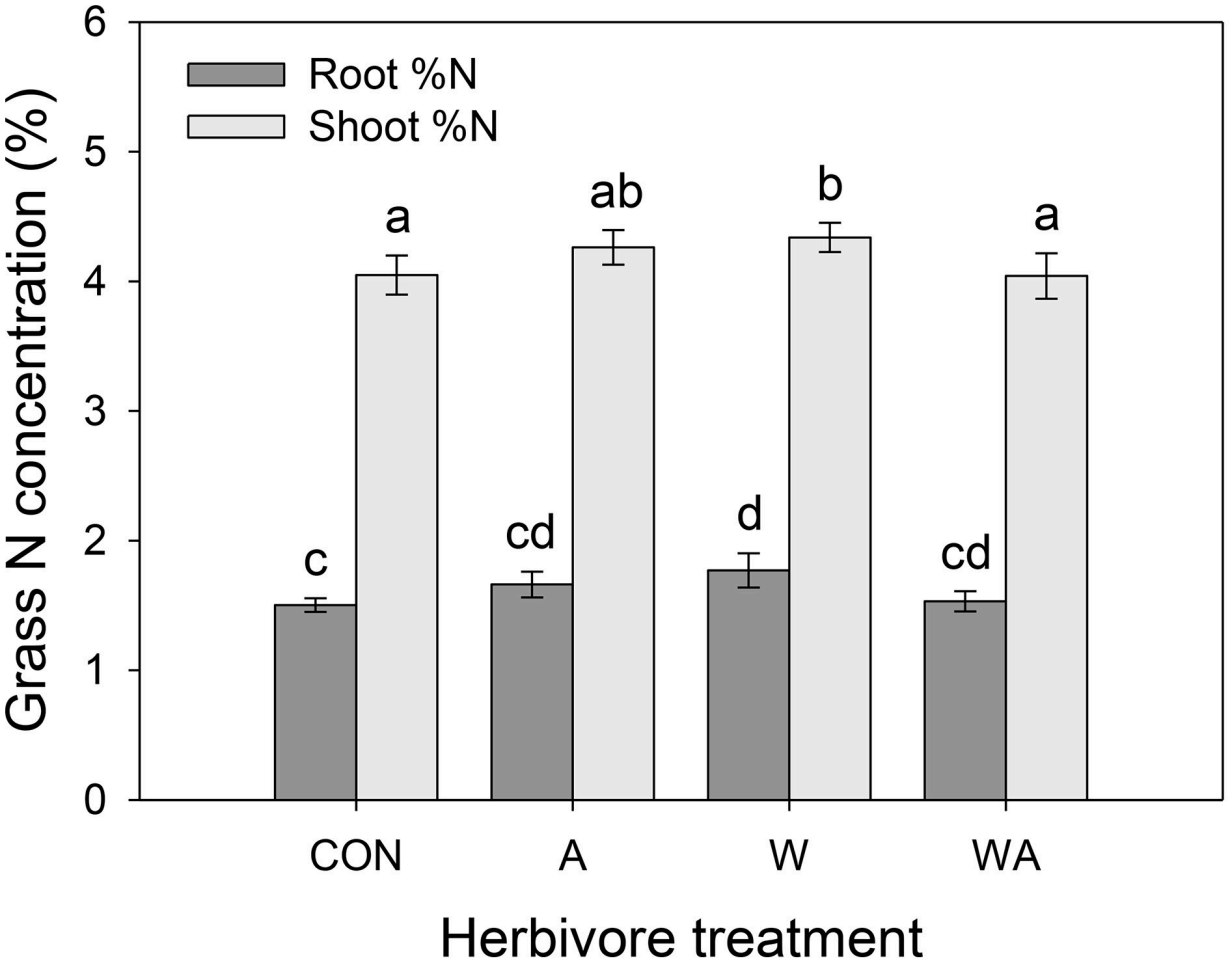
**The impacts of herbivore treatment on the root and shoot nitrogen concentrations of Harding grass**. CON were the control plants (no insects), A had aphids alone, W had weevils alone, and WA had both insects. Mean values (±SE) are shown. Bars with the same letters were not significantly different (*P* < 0.05).

Precipitation had no effect on grass root or shoot N concentrations, although shoot C concentrations decreased in grasses subjected to elevated precipitation compared with drought-stressed plants (Table 1; Figure S2A). Herbivore treatment significantly affected grass N concentrations, whereby plots inoculated with weevils alone contained grasses with higher root and shoot N concentrations compared with grasses in control plots (Figure 5).

**Figure 6:**
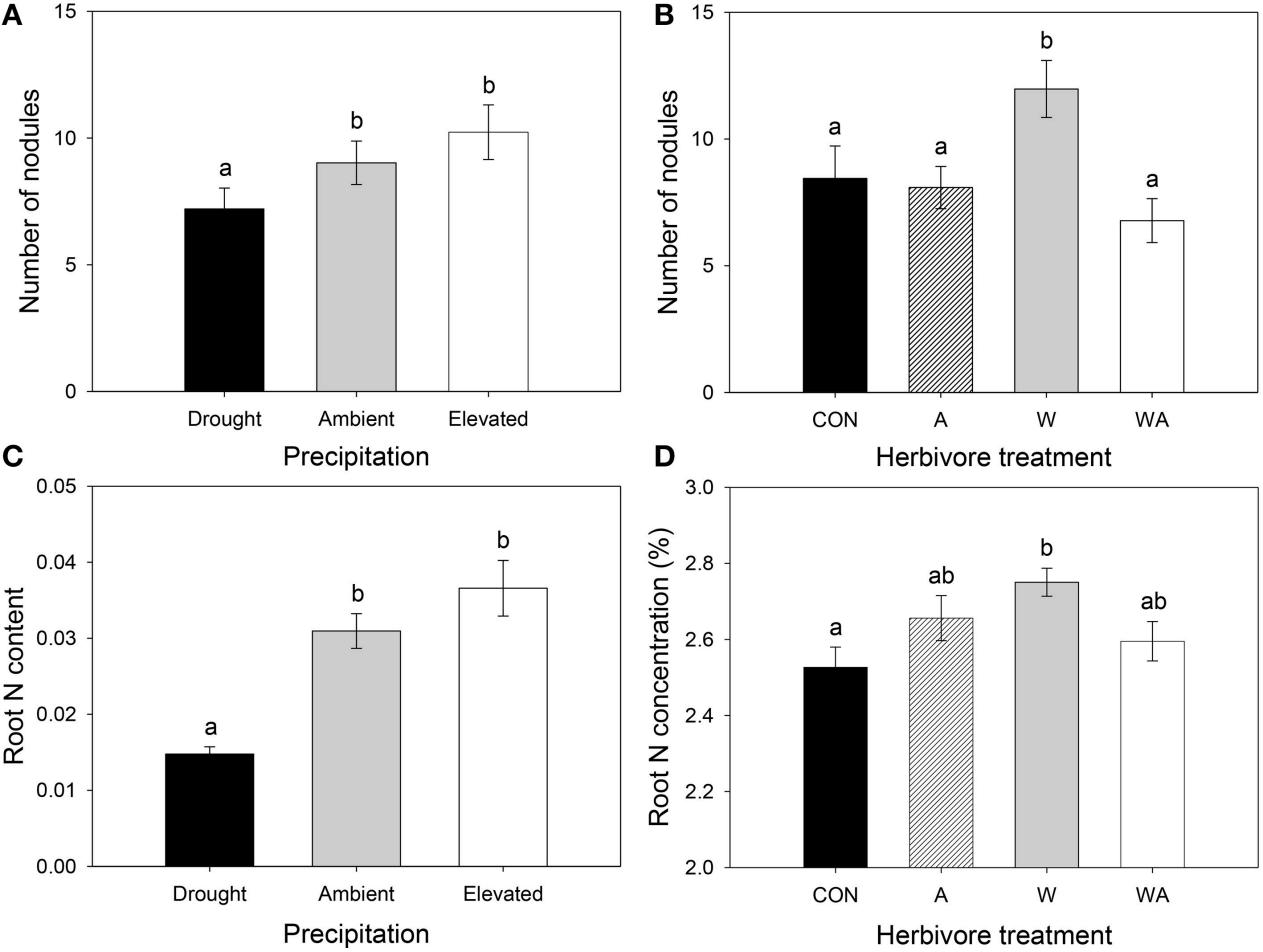
**The impacts of precipitation and herbivore treatment on lucerne nodulation (A,B) and the impacts of precipitation and herbivore treatment on root N content (C) and root N concentration (D), respectively**. CON were the control plants (no insects), A had aphids alone, W had weevils alone, and WA had both insects. Mean values (± SE) are shown. Bars with the same letters were not significantly different (*P* < 0.05).

Precipitation significantly affected the number of lucerne root nodules, with plants grown under drought containing, on average, 20 and 30% fewer nodules than those grown under ambient and elevated precipitation, respectively (Figure 6A). Herbivore treatment also significantly affected nodulation, whereby lucerne plants inoculated with weevils alone contained, on average, 48, 77, and 42% more root nodules than those containing aphids alone, those containing both insects and control plants, respectively (Figure 6B).

Roots of lucerne plants subjected to drought contained 52 and 60% less N than those under ambient and elevated precipitation, respectively (Figure 6C). Precipitation did not significantly impact the concentration of N in lucerne roots, although concentrations significantly increased in plants inoculated with weevils alone compared with control plants (Figure 6D). Precipitation interacted with herbivore treatment to significantly affect lucerne root C concentrations, with roots of plants inoculated with weevils alone containing a higher concentration of C than control plants, but only under drought conditions (Figure S2B).

### Amino acids

**Figure 7:**
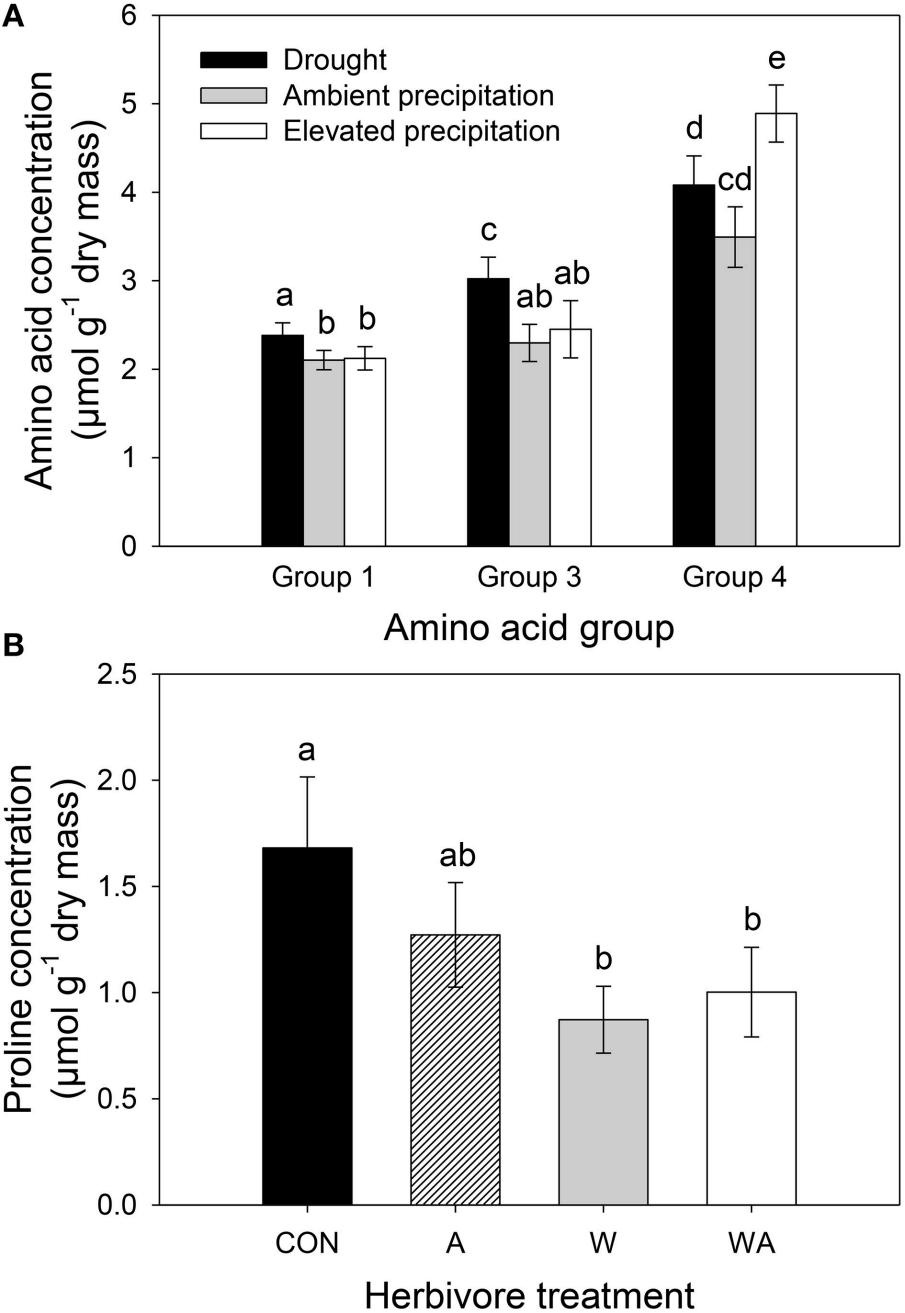
**The impacts of precipitation on individual groups of amino acids (A), namely group one (glycine, lysine, methionine, tyrosine, cysteine, histidine, arginine), group three (aspartate and glutamate), and group four (glutamine and serine) and the impacts of herbivore treatment on proline concentrations (B)**. CON were the control plants (no insects), A had aphids alone, W had weevils alone, and WA had both insects. Mean values (±SE) are shown. Bars with the same letters were not significantly different (*P* < 0.05).

Precipitation and herbivore treatment had no significant effect on total amino acid concentrations (Table S2) and PCA combining all amino acid concentrations revealed no separation between precipitation or herbivore treatments (Figure S3). Correlated amino acid groups (Figure S4) consisted of group one (glycine, lysine, methionine, tyrosine, cysteine, histidine, and arginine), group two (isoleucine, phenylalanine, leucine, threonine, and valine), group three (aspartate and glutamate), group four (glutamine and serine), and three independently varying amino acids (alanine, asparagine, and proline). PERMANOVA revealed a significant effect of precipitation on amino acid groups one, three, and four (Table S2). Specifically, concentrations of amino acid groups one and three increased in lucerne plants subjected to drought and concentrations of amino acid group four increased in those under elevated precipitation (Figure 7A). Herbivore treatment only affected one amino acid, with concentrations of proline significantly decreasing in lucerne plants that were inoculated with weevils (i.e., treatments W and WA; Figure 7B).

### Aphid responses

**Figure 8:**
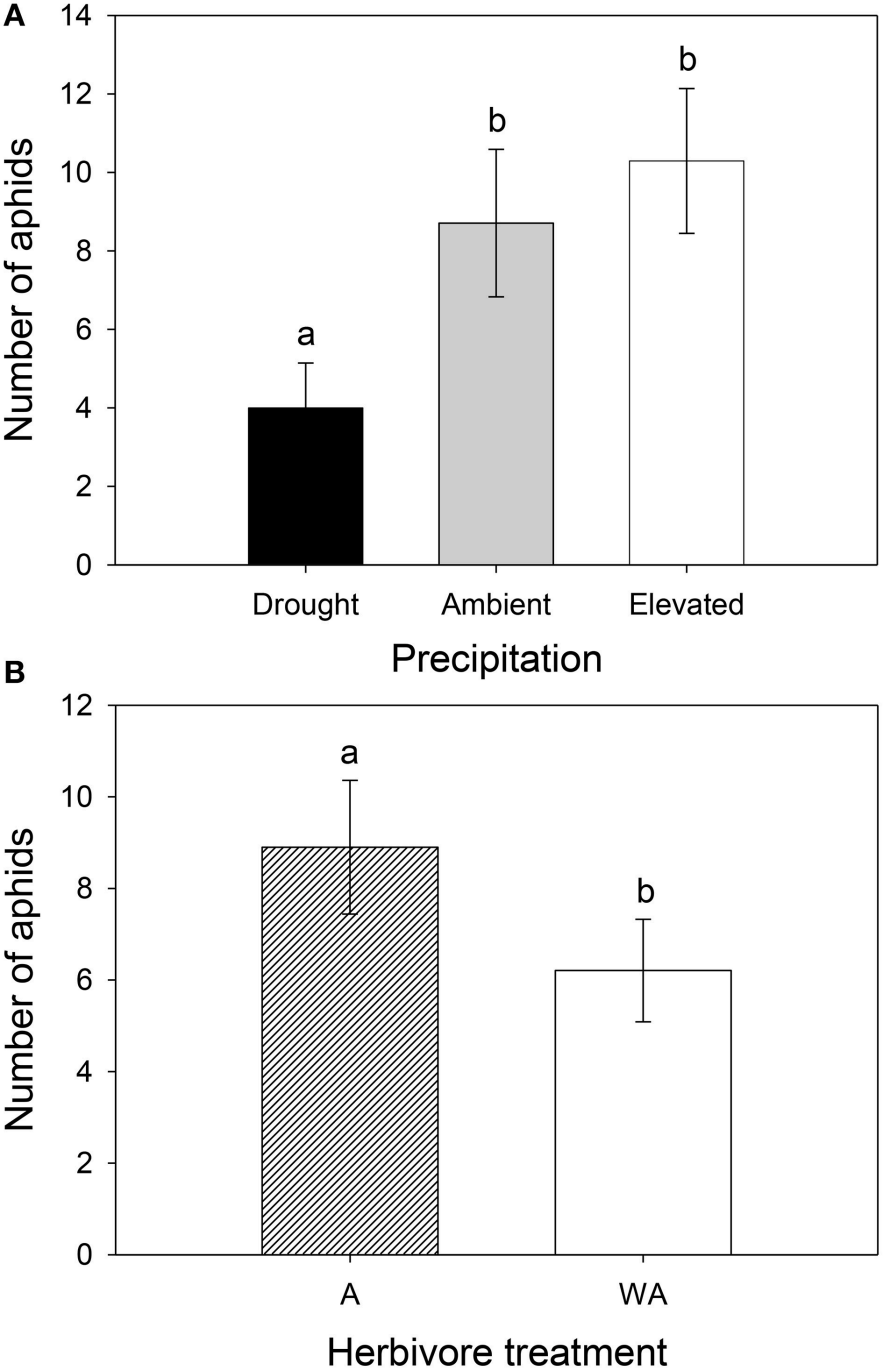
**The impacts of precipitation (A) and weevil presence (B) on the number of aphids**. A had aphids alone and WA had both weevils and aphids. Mean values (±SE) are shown. Bars with the same letters were not significantly different (*P* < 0.05).

Precipitation and herbivore treatment independently affected the number of aphids (Table 1). Lucerne plants subjected to drought supported 54 and 61% fewer aphids than those under ambient and elevated precipitation, respectively (Figure 8A). Additionally, aphid numbers decreased by 30% on lucerne plants inoculated with weevils compared to those with aphids alone (Figure 8B). Precipitation interacted with herbivore treatment to affect aphid colonization success, with weevils reducing aphid colonization success on plants under elevated precipitation. Drought generally reduced aphid colonization success, especially on plants with aphids alone (Figure S5).

**Figure 9:**
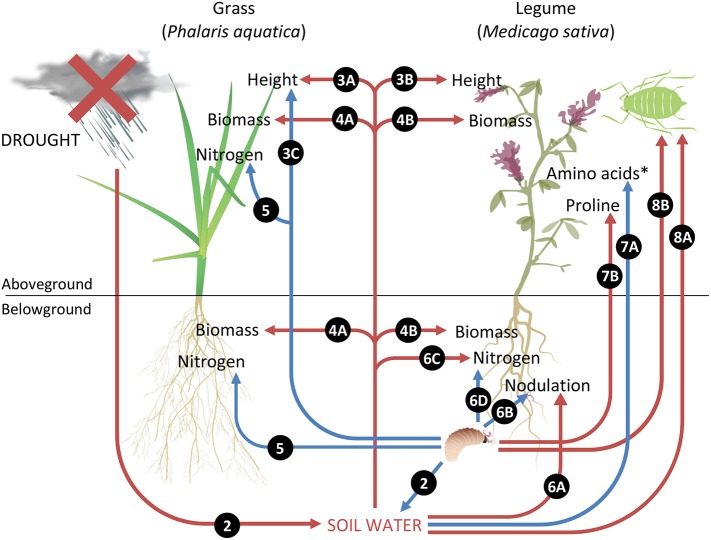
**Summary of key results illustrating plant–herbivore interactions under drought**. Red arrows indicate negative impacts and blue arrows represent positive impacts. Numbers refer to relevant figures associated with each effect. ^*^Amino acids represent concentrations of groups one and three amino acids, namely glycine, lysine, methionine, tyrosine, cysteine, histidine, arginine, aspartate, and glutamate.

The main findings are summarized in Figure 9, with numbers referring to relevant figures associated with each effect.

## Discussion

To our knowledge, this study is the first to investigate the combined effects of herbivory (both above- and below-ground) and climate change (i.e., drought and elevated precipitation) in a grass–legume system. The main results demonstrate that drought and weevil root herbivory have contrasting effects on plant growth and lucerne nodulation, whereas both factors reduced aphid population growth. These effects may scale-up to play important roles in governing ecosystem function.

### Soil water availability

Overall, drought reduced soil water content by 55% and elevated precipitation increased soil water content by 65% compared with ambient precipitation. Plots inoculated with weevils alone had increased soil water availability, suggesting that damage to nodules and roots by weevil larvae likely reduced water uptake by lucerne roots. Alternatively, burrowing activity of the weevils may have increased water penetration and reduced run-off, as observed in Johnson et al. (2015) with a soil conditioning ecosystem engineer (dung beetle; *Bubas bison* L.). The lack of an effect when aphids were feeding on lucerne simultaneously suggests that aphids counteracted the effects of weevils on soil water availability by either promoting soil water uptake or reducing weevil damage. In fact, aphid presence appeared to nullify the effects of weevils on multiple plant characteristics throughout this study, demonstrating the strong interactions between above- and below-ground stressors.

### Plant growth under water stress and herbivory

Drought clearly inhibited growth of both lucerne and Harding grass. Once water availability reaches an optimum level, more resources may be allocated to shoot growth, which likely explains why root biomass of both plant species did not vary significantly between ambient and elevated precipitation. Similarly, the ratio of roots to shoots decreased as water availability increased. This is consistent with the resource optimization hypothesis, which posits that plants will allocate fewer resources to their roots when water and nutrient availabilities are high (Gargallo-Garriga et al., 2014). Under drought, the biomass of lucerne and grass shoots decreased by 63 and 70%, respectively, relative to those under ambient precipitation. Grasses tend to be more sensitive to drought due to their shallower roots compared with the deeper taproots of lucerne (Hayes et al., 2012). While elevated precipitation increased plant growth in general, grasses dominated lucerne in plots subjected to elevated precipitation (i.e., shoot biomass increased by 37 and 72% in lucerne and grass, respectively, under elevated precipitation relative to ambient precipitation). Increased precipitation may therefore significantly disrupt the long-term ability of these plant species to co-exist (Tow, 1993; Tow et al., 1997). By harvest, weevil presence significantly increased grass height, most likely associated with the fertilizing effect of increased N leached from lacerated lucerne nodules. Alternatively, the increase in soil water availability for grasses in plots with weevils present alone may have been responsible for increasing the height of grasses, although that would not explain the concurrent increase in grass height when both insects were present. The significant effect of herbivory on grass height and tiller numbers, however, was not reflected in grass biomass.

### Impacts of water stress on C and N dynamics

When water is a limiting factor, metabolites involved with energy production and growth (especially carbohydrates) are often shifted from shoots to roots (Gargallo-Garriga et al., 2014), although drought may also lead to a decrease in the belowground C demand (Hasibeder et al., 2015). Drought effects on C allocation have also been found to be dependent on community composition. For example, Sanaullah et al. (2012) noted an increased allocation of C from shoots to roots under drought in monocultures of *Festuca arundinacea* (Schreb.), *L. perenne*, and *M. sativa*. When these three species were combined into a grass–legume mixture, however, C allocation to shoots increased when exposed to drought. The current study identified an increase in grass shoot C concentrations under drought compared with elevated precipitation, suggesting that Harding grass allocates more C to anti-stress mechanisms under drought (Peñuelas and Estiarte, 1998; Jentsch et al., 2011), although concentrations in drought-stressed grasses were not significantly higher than in those under ambient precipitation.

Drought reduced root N content and nodulation in lucerne plants, likely associated with decreased root N-fixation activity. The negative impact of drought stress on nitrogenase activity in leguminous plants is well-known, although the exact mechanisms remain unclear (Gil-Quintana et al., 2013). A decline in the concentration of root C suggests that the supply of reduced C to bacteria may limit nitrogenase activity and nodule development. Root N concentrations, however, did not decrease under drought, suggesting that drought-stressed lucerne plants were not N-deficient. Under drought conditions, root nodulation, and subsequently, nitrogen fixation activity may decline due to a lower demand for N to support growth (Streeter, 2003). Predicting the responses of legumes to environmental change will be particularly important to maintain the N dynamics within the many terrestrial systems that are currently dominated by inputs of fixed N by legumes (Whitehead, 2000; Lilley et al., 2001; Peoples and Baldock, 2001; Angus and Peoples, 2012).

### Impacts of weevils on C and N dynamics

Lucerne nodulation and root N concentrations increased in response to weevil herbivory, suggesting overcompensatory growth in response to nodule damage. Other studies have also shown nodule overcompensation in lucerne after root feeding by *Sitona hispidulus* Fabricius (Quinn and Hall, 1992) and *S. discoideus* (Ryalls et al., 2013b). No significant increase, however, was observed when plants were inoculated with both insects. When both weevil larvae and aphids are feeding on the plant, root recovery, and stress-related increases in plant nutrients or the diversion of resources above- or below-ground may be less likely because the plant remains in a constant state of stress (Ryalls et al., 2015). Weevils mitigated the effects of drought on lucerne root C concentrations by increasing root C concentrations. This is surprising considering that drought would most likely decrease weevil damage associated with declines in larval numbers (Goldson et al., 1986; Johnson et al., 2010). The difficulty in extracting *S. discoideus* larvae from the soil (Wightman, 1986) made it impossible to corroborate this assumption but additional studies using a more conspicuous species (e.g., whitefringed weevil, *Naupactus leucoloma* Boh.) may benefit from counting and weighing larvae.

Grass root and shoot N concentrations increased in plots that were inoculated with weevils alone, suggesting that larval damage to lucerne roots and nodules caused N leakage into the soil, which subsequently increased the uptake of N by Harding grass (Bardgett et al., 1999; Murray et al., 2013). This information may be particularly useful for ecosystems that suffer from legume-feeding pests or processes that damage legume roots, with consequences for productivity, nutrient balance, and species richness within ecological communities. Murray and Clements (1998) similarly identified an increase in N in wheat (*Triticum arvense* L.) from weevil-infested white clover. Other studies have identified a transfer of N from white clover to perennial ryegrass in response to herbivory by root-feeding nematodes (Bardgett et al., 1999; Dromph et al., 2006). In contrast, Ayres et al. (2007) noted a 13% reduction in N transfer from white clover to perennial ryegrass when clover roots were damaged by nematodes. They also identified a significant increase in grass root N when clover was subjected to simulated defoliation (through clipping). In this case, *S. discoideus* adults are defoliating insects so simultaneous feeding of larvae and adults may lead to even greater increases in N transferred to grasses. Increasing community complexity can alter the nutrient balance of plants differently, even if one plant (in this case, Harding grass) is not directly impacted. Given the prevalence of N-limitation in terrestrial ecosystems (Vitousek and Howarth, 1991), increased N supply has the potential to influence plant productivity, especially in grassland ecosystems (Ayres et al., 2007).

### Amino acid and aphid responses to water stress and weevils

Sustained water stress in plants tends to affect aphids negatively (Huberty and Denno, 2004). *A. pisum* densities, in particular, have been closely linked to lucerne water content, with populations suffering lower growth rates when lucerne is drought stressed (Forbes et al., 2005). In the current study, drought negatively impacted aphids, although amino acid concentrations generally increased in plants under drought compared with those under ambient precipitation. Hale et al. (2003) also reported a reduction in aphid (*Rhopalosiphum padi* L.) performance alongside an increase in amino acid concentrations in plants subjected to drought, suggesting that lower sap ingestion rates on drought-stressed plants are responsible for reducing aphid performance overall and override any positive effects on amino acid concentrations. From a community perspective, drought may somewhat alleviate the impacts of sap-feeding herbivores on plants.

Many studies have demonstrated how root feeders can influence aphid populations through plant-mediated mechanisms. Generally, aboveground aphids are positively affected by belowground root feeders (Johnson et al., 2012), although species that feed on legumes should be considered separately from those that feed on non-leguminous plants since legume root herbivores feed directly on the sites of N fixation and are more likely to negatively impact aphids by impairing root function and lowering the quality of phloem sap (Goldson S. L. et al., 1988; Ryalls et al., 2013b, 2015). Here, proline concentrations decreased when weevils were present, which may have contributed to the reduction in aphid numbers when weevils were feeding on the plant simultaneously. Proline concentrations in plants that were inoculated with aphids alone, however, were not significantly lower than concentrations in plants that were inoculated with both insects. Aphids may have mitigated the negative effects of weevil nodule feeding on foliar amino acid concentrations by promoting the metabolism of amino acids in lucerne (Guo et al., 2013) and masking the decrease in proline caused by weevils. While weevil larvae increased nodulation in lucerne, the effects on aphids may not have been apparent by the end of the experiment. Uncertainty exists as to whether the stimulation of nodulation over a longer timeframe would actually have a positive impact on aphids considering the dependency of aphids on plant nitrogen (Butler et al., 2012). Incorporating weevil larval performance data and quantifying root damage would provide useful insights into damage intensity and thresholds in lucerne, as described by Goldson et al. (1987; 1988) in New Zealand. Moreover, these data would allow us to examine aboveground–belowground interactions in the opposite direction (i.e., the impacts of shoot herbivory on root herbivores). No clear trend has yet emerged from studies analysing the effects of shoot herbivory on root herbivores (Johnson et al., in press and references therein), although it is uncertain whether patterns in lucerne or other legumes would emerge if these data were available. Understanding the complex interactions between above- and below-ground communities and how they fluctuate over time is essential for characterizing the fundamental mechanisms driving plant community structure (De Deyn and Van der Putten, 2005; De Deyn et al., 2007).

### Conclusions

Global climate change and herbivory can modify the transfer of N and shape the competitive interactions between legumes and non-N-fixing grasses, with important implications for plant community structure. The results demonstrate how legume nodule herbivory by weevil larvae can increase N uptake and productivity of a companion grass species and decrease aphid populations via changes in individual amino acid concentrations. Moreover, drought decreased plant productivity in general and reduced aphid populations on lucerne, potentially via reduced phloem turgor pressure. With the frequency and length of droughts projected to increase under global climate change (Trenberth et al., 2003; IPCC, 2007; Gargallo-Garriga et al., 2014), understanding how these factors can shape plant susceptibility to insect pests and maintain the balance between an efficient grass–legume mixture is clearly a priority for achieving food security (Ayres et al., 2007; Gregory et al., 2009; Boschma et al., 2010). This will be especially important in terrestrial ecosystems that rely more on mineralization as a source of N for grasslands and other plant communities in the future (Newbould, 1989; Angus et al., 2006; Grace et al., 2015). Ultimately, these data inform adaptation strategies aimed at relieving the disruption caused by insect herbivore pests. With the threats of climate change on ecological communities apparent, combining multiple interacting species in environmental studies is key to maintaining ecosystem services and protecting our food resources, especially as human population levels climb toward 10 billion by the end of the century (Lal, 2006; Birch et al., 2011).

## Author contributions

JR and SJ conceived and designed the experiment. JR collected the field data. JR conducted chemical analyses and analyzed the data. JR wrote the paper with assistance from SJ, BM, and MR.

## Funding

Ph.D. Scholarship funded by the Hawkesbury Institute for the Environment (Western Sydney University).

### Conflict of interest statement

The authors declare that the research was conducted in the absence of any commercial or financial relationships that could be construed as a potential conflict of interest.
