# Supplementary material for: Above–Belowground Herbivore Interactions in Mixed Plant Communities Are Influenced by Altered Precipitation Patterns
Source: Front Plant Sci. 2016 Mar 23;7:345. doi: 10.3389/fpls.2016.00345 (PMC4804199; doi:10.3389/fpls.2016.00345)
Supplement: Supplementary file 1 [file DataSheet1.docx]

**Supplementary material**

**Table S1.** Mixed model results combining height data for lucerne and Harding grass over time (accounting for repeated measures by including week as a fixed effect).

|  | **Grass**  **(*Phalaris aquatica*)** | | |  | **Lucerne**  **(*Medicago sativa*)** | | |
| --- | --- | --- | --- | --- | --- | --- | --- |
| **Factor** | ***df*** | ***F*** | ***P*** |  | ***df*** | ***F*** | ***P*** |
| Herbivore treatment | 3,45 | 6.63 | <0.001 |  | 3,45 | 1.13 | 0.347 |
| Precipitation | 2,15 | 70.06 | <0.001 |  | 2,15 | 15.51 | <0.001 |
| Week | 2,120 | 321.96 | <0.001 |  | 2,264 | 707.16 | <0.001 |
| Herbivore treatment × Precipitation | 6,45 | 1.65 | 0.156 |  | 6,45 | 0.42 | 0.865 |
| Herbivore treatment × Week | 6,120 | 2.23 | 0.045 |  | 6,264 | 0.54 | 0.776 |
| Precipitation × Week | 4,120 | 80.67 | <0.001 |  | 4,264 | 100.52 | <0.001 |
| Herbivore treatment × Precipitation  × Week | 12,120 | 0.35 | 0.978 |  | 12,264 | 0.88 | 0.573 |

**Table S2.** Results from multivariate permutational analysis (PERMANOVA) of herbivore treatment (Treatment) and precipitation effects on different groups of correlated amino acids. A statistically significant effect is indicated by *(*P* < 0.05).

|  | **df** | **SS** | **MS** | **Pseudo-F** | **P(perm)** |
| --- | --- | --- | --- | --- | --- |
| **All amino acids** |  |  |  |  |  |
| Treatment | 3,60 | 0.107 | 0.036 | 0.737 | 0.716 |
| Precipitation | 2,60 | 0.187 | 0.093 | 1.928 | 0.065 |
| Treatment × Precipitation | 6,60 | 0.091 | 0.015 | 0.312 | 1.000 |
| **Group 1 (Glycine, Lysine, Methionine, Tyrosine, Cysteine, Histidine, Arginine)** | | | | | |
| Treatment | 3,60 | 0.104 | 0.035 | 0.796 | 0.646 |
| Precipitation | 2,60 | 0.192 | 0.096 | 2.205 | 0.024* |
| Treatment × Precipitation | 6,60 | 0.183 | 0.031 | 0.702 | 0.858 |
| **Group 2 (Isoleucine, Phenylalanine, Leucine, Threonine, Valine)** | | | | | |
| Treatment | 3,60 | 0.039 | 0.013 | 0.485 | 0.741 |
| Precipitation | 2,60 | 0.038 | 0.019 | 0.715 | 0.509 |
| Treatment × Precipitation | 6,60 | 0.034 | 0.006 | 0.213 | 0.994 |
| **Group 3 (Aspartate, Glutamate)** | | | | | |
| Treatment | 3,60 | 0.151 | 0.050 | 1.165 | 0.306 |
| Precipitation | 2,60 | 0.239 | 0.120 | 2.762 | 0.039* |
| Treatment × Precipitation | 6,60 | 0.134 | 0.022 | 0.516 | 0.871 |
| **Group 4 (Glutamine, Serine)** | | | | | |
| Treatment | 3,60 | 0.107 | 0.036 | 0.738 | 0.625 |
| Precipitation | 2,60 | 0.308 | 0.154 | 3.176 | 0.019* |
| Treatment × Precipitation | 6,60 | 0.128 | 0.021 | 0.439 | 0.937 |
| **Alanine** | | | | | |
| Treatment | 3,60 | 0.099 | 0.033 | 1.177 | 0.325 |
| Precipitation | 2,60 | 0.026 | 0.013 | 0.457 | 0.685 |
| Treatment × Precipitation | 6,60 | 0.178 | 0.030 | 1.058 | 0.392 |
| **Asparagine** | | | | | |
| Treatment | 3,60 | 0.150 | 0.050 | 0.591 | 0.708 |
| Precipitation | 2,60 | 0.385 | 0.192 | 2.277 | 0.087 |
| Treatment × Precipitation | 6,60 | 0.134 | 0.022 | 0.264 | 0.995 |
| **Proline** | | | | | |
| Treatment | 3,60 | 0.551 | 0.184 | 2.484 | 0.027* |
| Precipitation | 2,60 | 0.022 | 0.011 | 0.146 | 0.960 |
| Treatment × Precipitation | 6,60 | 0.345 | 0.057 | 0.777 | 0.621 |

**Figure S1.** The impacts of precipitation (A) and weevil presence (B) on the number of grass tillers. CON are the control plants (no insects), A have aphids alone, W have weevils alone and WA have both insects. Mean values ± standard errors are shown. Bars with the same letters were not significantly different (*P* < 0.05).

**Figure S2.** The impacts of precipitation on the root and shoot carbon concentrations of Harding grass (A) and the interactive effects of precipitation and herbivore treatment on the root carbon concentrations of lucerne (B). Mean values ± standard errors are shown. Bars with the same letters were not significantly different (*P* < 0.05).


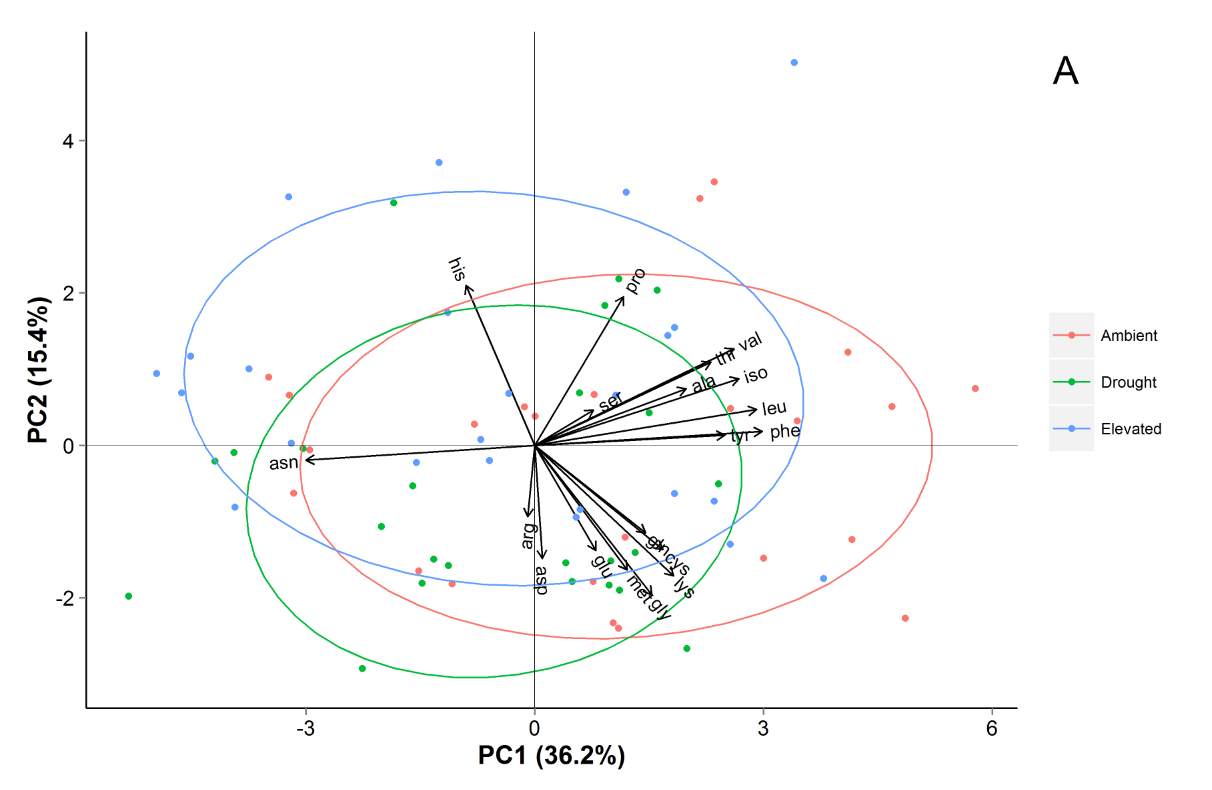


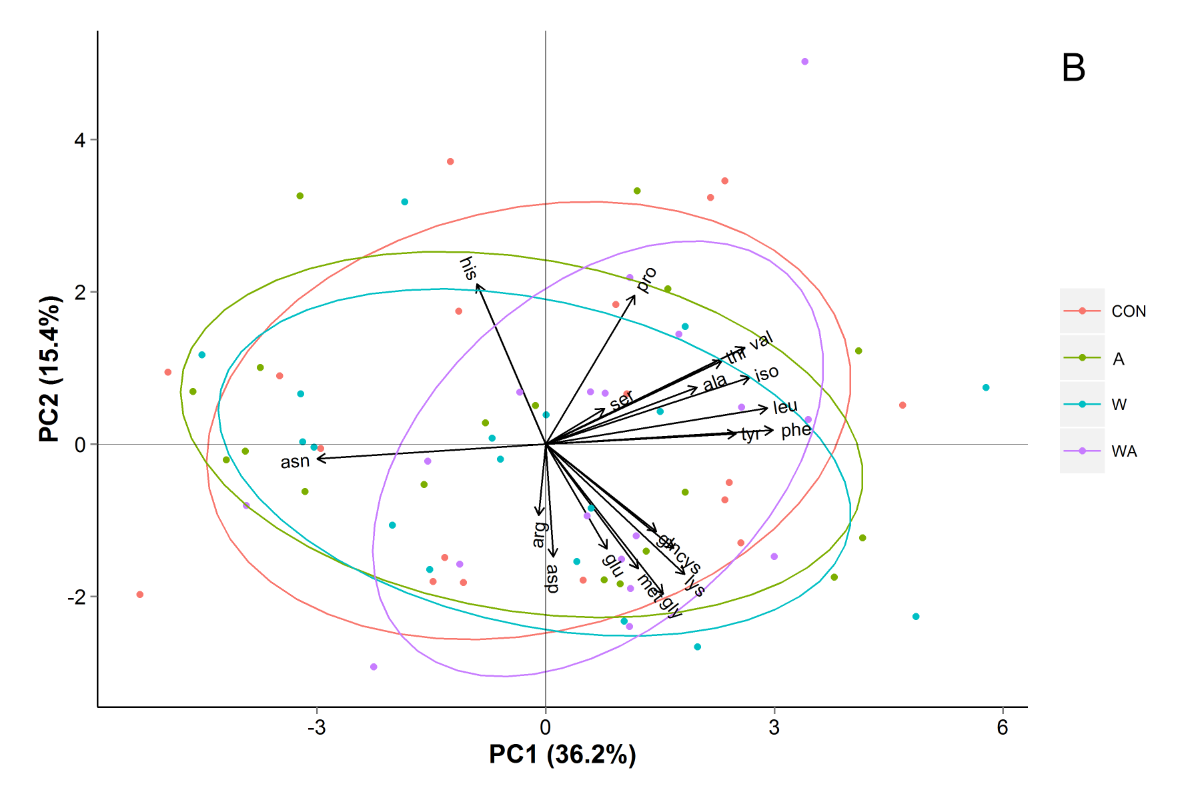


**Figure S3.** Principal component analysis of foliar amino acid concentration data with attribute loadings on the first two components PC1 and PC2. Amino acid abbreviations are denoted in Figure S4. Plots and ellipses (representing 68% of the predicted data) were coloured according to precipitation (A) and herbivore treatments (B). CON are the control plants (no insects), A have aphids alone, W have weevils alone and WA have both insects.

**
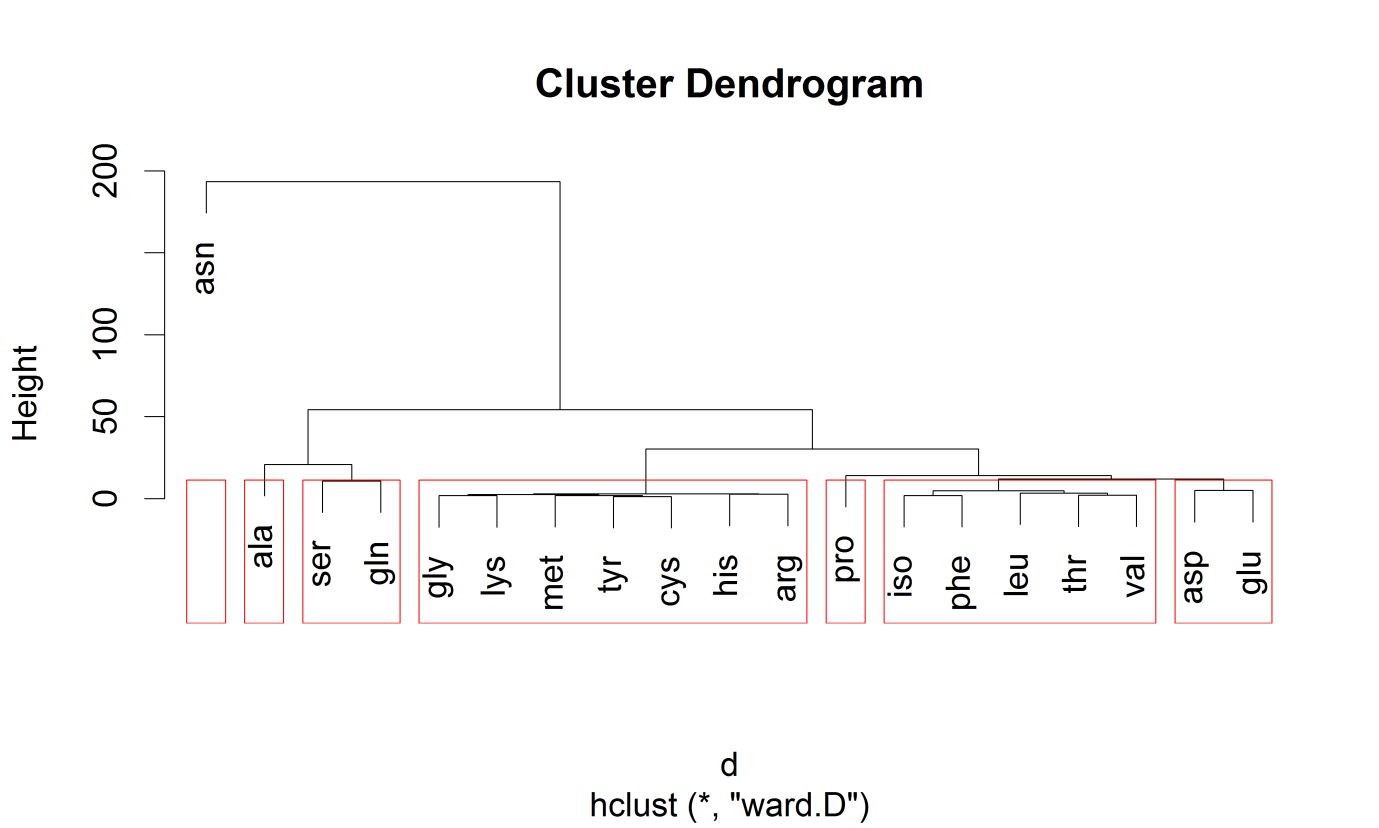
**

**Figure S4.** Hierarchical tree of clustered amino acids. Grouped amino acids were all correlated (>0.7). Amino acid abbreviations are as follows: asparagine (asp), alanine (ala), serine (ser), glutamine (gln), glycine (gly), lysine (lys), methionine (met), tyrosine (tyr), cysteine (cys), histidine (his), arginine (arg), proline (pro), isoleucine (iso), phenylalanine (phe), leucine (leu), threonine (thr), valine (val), aspartate (asp) and glutamate (glu).

**Figure S5.** The interactive effects of precipitation and weevil presence on the percentage of plants colonised by aphids (± standard errors). Bars with the same letters were not significantly different (*P* < 0.05).
